# Antepartum urinary tract infection and postpartum depression in Taiwan – a nationwide population-based study

**DOI:** 10.1186/s12884-018-1692-6

**Published:** 2018-03-27

**Authors:** Jui-Ming Liu, Feng-Hsiang Chiu, Yueh-Ping Liu, Shu-Pin Chen, Hsun-Hao Chan, Jing-Jung Yang, Fung-Wei Chang, Ren-Jun Hsu

**Affiliations:** 1 Division of Urology, Department of Surgery, Taoyuan General Hospital, Ministry of Health and Welfare, Taoyuan, Taiwan; 2 Department of Medicine, National Yang-Ming University, Taipei, Taiwan; 3 Graduate Institute of Life Sciences, National Defense Medical Center, Taipei, Taiwan; 4 American-Sino Women’s & Children’s, Hospital (SongYuan), Shanghai City, China; 5 Lihuili Eastern Hospital, Ningbo Medical Center, Ningbo City, Zhejiang province China; 6 Department of emergency Medicine, National Taiwan University Hospital, Taipei, Taiwan; 7 Division of Genetics and endocrinology, Chang Gung Children’s Hospital, Taoyuan, Taiwan; 8 Division of Urology, Department of Surgery, Yumin medical corporation Yumin hospital, Nantou, Taiwan; 9 Department of Psychiatry, Cardinal Tien Hospital, New Taipei City, Taiwan; 10 Institute of Biomedical Engineering, College of Medicine and College of Engineering, National Taiwan University, Taipei, Taiwan; 11 Superintendent, Tri-Service General Hospital Penghu Branch, National Defence Medical Center, Penghu Branch, Taiwan; 12 Biobank Management Center of the Tri-Service General Hospital, National Defense Medical Center, Taipei, Taiwan; 13 Department of Pathology and Graduate Institute of Pathology and Parasitology, the Tri-Service General Hospital, National Defense Medical Center, No. 161, Sec. 6, Minquan E. Road, Neihu District, Taipei, 114 Taiwan

**Keywords:** Urinary tract infection, National health insurance research database, Postpartum depression, Female

## Abstract

**Background:**

Urinary tract infections (UTIs) are among the most common bacterial infections in pregnant women due to anatomic and physiologic changes in the female urinary tract during pregnancy, and antepartum UTIs can cause adverse pregnancy outcomes that may induce mental stress. There have only been a few studies, however, investigating antepartum UTIs and mental stress. As such, the present study was conducted in order to investigate the association between antepartum UTIs and postpartum depression (PPD).

**Methods:**

We used data from the 2000–2013 National Health Insurance Research Database (NHIRD) of Taiwan. Data regarding a total of 55,087 singleton pregnancies was utilized, including data regarding 406 women who were newly diagnosed with PPD in the first 6 months postpartum. The associations between PPD and antepartum UTIs or other risk factors were examined by multiple logistic regression analysis.

**Results:**

The logistic regression analysis results indicated that PPD was associated with antepartum UTIs (adjusted odds ratio [aOR] 1.27; 95% confidence interval [CI] (1.07–1.65). Furthermore, the risk of PPD was higher in women with an upper antepartum UTI (aOR 2.97 (1.31, 6.77) than in those with a lower antepartum UTI (aOR 1.21 (1.02, 1.58)).

**Conclusions:**

Antepartum UTIs, particularly upper antepartum UTIs, are significantly associated with PPD. This information may encourage physicians to pay greater attention to the mental health of women who have suffered upper UTIs during their pregnancies.

## Background

Previous epidemiological studies have reported that postpartum depression (PPD) occurs in approximately 10–15% of women after delivery [1–3]. PPD is defined in the fifth edition of the Diagnostic and Statistical Manual of Mental Disorders (DSM-V) [4] as a major depressive episode occurring during pregnancy or in the 4 weeks following delivery. However, PPD usually occurs in the first 6 months after delivery [5, 6]. A recent study of postnatal women in the UK and Taiwan showed that 19% of Taiwanese women and 18% of British women reported experiencing PPD [7].

During pregnancy, women experience substantial physiological and psychological changes. These changes may lead postpartum depressive mood. Previous studies have highlighted numerous risk factors associated with PPD including physical, psychological, obstetric, pediatric, socio-demographic, and cultural factors [8, 9].

Pregnancy also causes anatomic and physiologic changes to the female urinary tract. Urinary tract infections (UTIs) are among the most common bacterial infections during pregnancy [10], with approximately 2–10% of pregnant women experiencing a UTI [11]. Antepartum UTIs are associated with some adverse pregnancy outcomes [12] and are also one of the major causes of antepartum admissions [13].

To the best of our knowledge, however, there have been very few reports on the relationship, if any, between antepartum UTIs and PPD. As such, the present population-based study sought to (1) determine whether there is a relationship between antepartum UTIs and PPD and (2) determine whether different locations of antepartum UTIs (that is, in the upper or lower urinary tract) result in different effects on the mental health of pregnant women.

## Methods

### Data source and collection

We used the National Health Insurance Research Database (NHIRD) for this population-based study. The NHIRD is a database collected by the National Health Insurance (NHI) program of Taiwan, which is a unique health insurance system covering almost all the citizens of Taiwan. The NHI program was begun in 1995 and provided coverage for 99.9% of the 23 million people in Taiwan as of 2013 [14]. The NHIRD is a large-scale database that contains outpatient and inpatient medical information about the patients included in the database, including information on their medication use, surgical procedures, intervention procedures, and clinical prescriptions [15, 16]. At present, there are more than 2000 published studies that have utilized data included in this database [17]. The present retrospective population-based study used a sub-dataset of the NHIRD called the Longitudinal Health Insurance Database 2000 (LHID 2000) that included data from January 2000 to December 2013. The LHID 2000 contains 1 million people who were randomly selected from the total of 23 million residents included in the NHIRD in 2000 [18, 19]. The disease diagnoses of the people included in the database were made according to the International Classification of Diseases, 9th revision, Clinical Modification (ICD-9-CM).

### Study population

**Fig. 1:**
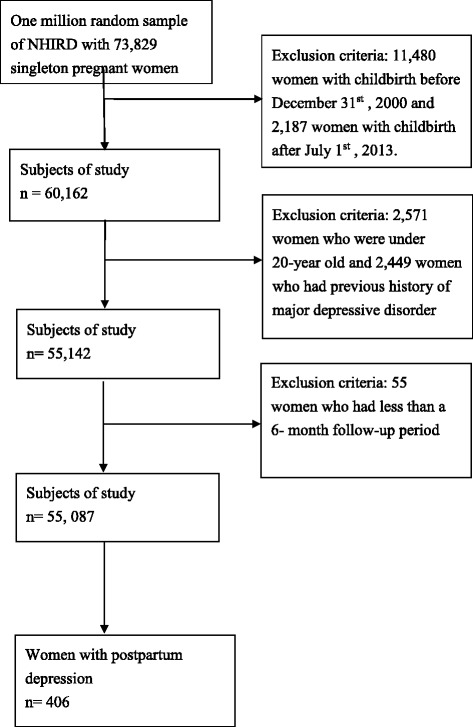
Flowchart of the collection of subjects from the Longitudinal Health Insurance Database2000 (LHID2000), a sub-dataset of National Health Insurance Research Database (NHIRD), from January 2000 to December 2013

The study population was selected from the LHID 2000 covering the period from January 2000 to December 2013. The selection of participants in this study is shown in Fig. 1. There were 73,829 singleton pregnancies among the database cohort during the study period. The exclusion criteria were as follows (1) women with delivery before Dec 31st 2000 or after July 31st 2013 (*n* = 11,480); (2) women who were younger than 20 years old (*n* = 2571); (3) women who had a previous history of major depressive disorder (*n* = 2449); and (4) women with a follow-up period of less than 6 months (*n* = 55). In Taiwanese civil law, people in their twenty years of age are adult. We chose adult women in our study with approval by the Institutional Review Board. We selected women identified as having PPD according to a medical record of a PPD diagnosis made by a psychiatrist and by the use of antidepressants in the first 6 months after delivery (ICD-9-CM: 309, 311, 296.2, 296.3, 296.5, 300.4).

Each UTI was diagnosed by collecting a clean-voided, midstream urine sample for urinalysis and urine culture. A positive urine culture was defined by the isolation of at least one single microorganism with > 10,000 colonies/mL. An upper UTI was diagnosed according to physical examination, urinalysis, and urine culture results, with positive physical examination findings including flank pain, fever, chills, anorexia, nausea, and vomiting. The diagnosis of a lower UTI was also made via physical examination, urinalysis, and urine culture results, with the typical manifestations of a lower UTI including dysuria, frequency, urgency, and suprapubic pain.

### Variables

Variables were selected based on clinical manifestations or complications during pregnancy. We retrospectively investigated different variables in women with or without PPD. In this study, antepartum UTIs were grouped into two categories: upper and lower UTIs. An upper UTI consists of an infection of the renal parenchyma and pelvicaliceal system. This includes cases of pyelonephritis, pyelitis, and perirenal infection or abscess (ICD-9-CM: 590.1, 590.2, 590.8, 590.9). Lower UTI infections are located in the lower urinary tract and include cystitis and urethritis (ICD-9-CM: 595.0, 595.1, 595.2, 595.3, 595.4, 595.8, 595.9, 597.8, 599.0). Additional factors of antepartum, peripartum, and postpartum complications that are related to PPD, according to a previous study [20], were also analyzed, including antepartum hemorrhage (ICD-9-CM: 641.8, 641.9), eclampsia or pre-eclampsia (ICD-9-CM: 642.4, 642.5, 642.6), premature separation of placenta (ICD-9-CM: 641.2), placenta previa (ICD-9-CM: 641.0, 641.1), oligohydramnios (ICD-9-CM: 658), polyhydramnios (ICD-9-CM: 657), poor fetal growth (ICD-9-CM: 656.5), excessive fetal growth (ICD-9-CM: 656.6), cervical incompetence (ICD-9-CM: 654.5), premature birth (ICD-9-CM: 644), chronic pulmonary disease (ICD-9-CM: 490–492, 494,496), hypertension (ICD-9-CM: 401.1401.9), hypertension-complicated pregnancy (ICD-9-CM: 642.0, 642.1, 642.2, 642.3, 642.9), diabetes complicating pregnancy childbirth (ICD-9-CM: 648.0), hyperlipidemia (ICD-9-CM: 272.4), heart disease (ICD-9-CM: 393–398, 402, 404.0, 404.1, 404.9, 410–414, 415.0, 416.1, 416.8, 416.9, 420–429), anemia (ICD-9- CM: 280–285), cerebrovascular disease (ICD-9-CM: 430–438), Parkinson disease (ICD-9-CM: 332), epilepsy (ICD-9-CM: 345), tuberculosis (ICD-9-CM: 011,012), asthma (ICD-9-CM: 493), chronic kidney disease (ICD-9-CM: 585,586,588). The monthly incomes of the study population, as per insurance information, were divided into four groups: < 20,000, 20,000–40,000, 40,000–60,000, and ≥60,000 new Taiwan dollars [21].

### Statistical analysis

We used the Microsoft® SQL Server® 2008 to calculate and manage the patients’ baseline data. The IBM SPSS statistics software v20 (IBM SPSS, 2013) was used for further data analysis. All the variables were calculated as percentages. Standard deviations and mean values were determined for the quantitative variables. Statistical significance was determined using Student’s T test and the Chi-square test. Multivariate logistic regression analysis was used to determine any associations between antepartum UTIs, PPD, and additional factors. We selected risk factors into further multivariate logistic regression analysis by which total n≧5 in the PPD group. A two-sided *p*-value < 0.05 was regarded as statistically significant in all statistical tests.

## Results

### Demographics

Data for a total of 55,087 women who had singleton pregnancies between January 2001 and June 2013 was utilized in this study, including data for a total of 406 women who were newly diagnosed with PPD in the first 6 months postpartum. All the singleton pregnancy women were divided into two groups: a PPD group (*n* = 406) and a non-PPD group (*n* = 54,691). Table 1 shows the demographic characteristics of the two study groups. Women with PPD had older ages (31.9 ± 10.4 years, *p* < 0.001) compared with the non-PPD group. There were many antepartum comorbidities that were found to be significantly associated with PPD, including chronic pulmonary disease, hypertension, peptic ulcer disease, chronic kidney disease, liver cirrhosis, hypertension-complicated pregnancy, heart disease, asthma, epilepsy, early delivery onset, premature birth, and UTI. Both upper and lower tract UTIs showed significant associations with PPD. The average hospital stay of all the women for childbirth was 4.8 ± 2.2 days. The average time to PPD diagnosis was 88.0 ± 51.0 days after delivery.

**Table 1:** Demographic characteristics of study populations in Taiwan from 2000 to 2013

Demographic factor	Total pregnant women *n* (%)	Without PPD *n* (%)	PPD *n* (%)	*P* value^a^
No. of patients	55,087	54,681	406	
Age (mean ± standard deviation, years)	30 ± 6.2	29.9 ± 6.1	31.9 ± 10.4	0.003
Age_group				< 0.001^**^
20–24	7986(14.5)	7928(14.5)	58(14.3)	
25–29	19,548(35.5)	19,405(35.5)	143(35.2)	
30–34	19,092(34.7)	18,968(34.7)	124(30.5)	
35–39	6724(12.2)	6679(12.2)	45(11.1)	
≧40	1737(3.1)	1701(3.1)	36(8.9)	
Hospital stay (mean ± standard deviation)	4.8 ± 2.2	4.8 ± 1.6	4.2 ± 3.2	
Monthly income (TWD ^b^)				< 0.05^*^
< 20,000	36,586(66.4)	36,292(66.4)	294(72.4)	
20,000–40,000	14,340(26.0)	14,246(26.1)	94(23.2)	
40,000–60,000	3687(6.7)	3670(6.6)	17(4.2)	
≧60,000	474(0.9)	473(0.9)	1(0.2)	
Antepartum urinary tract infection				
Yes	7325(13.3)	7255(13.3)	70(17.2)	< 0.05^*^
Upper	255(0.5)	249(0.5)	6(1.5)	< 0.05^*^
Lower	7070(12.9)	7006(12.9)	64(16)	< 0.05^*^
No	47,762(86.7)	47,426(86.7)	336(82.8)	
Co-morbidity disease				
Antepartum hemorrhage	460(0.8)	456(0.8)	4(1.0)	0.739
Premature separation of placenta	320(0.6)	316(0.6)	4(1.0)	0.282
Placenta previa	854(1.6)	849(1.6)	5(1.2)	0.602
Eclampsia or pre-eclampsia	799(1.5)	792(1.4)	7(1.7)	0.643
Unstable lie	47(0.1)	47(0.1)	0	–
Polyhydramnios	37(0.1)	37(0.1)	0	–
Oligohydramnios	4118(7.5)	4082(7.5)	36(8.9)	0.285
Poor fetal growth	521(0.9)	518(0.9)	3(0.7)	0.666
Excessive fetal growth	532(1.0)	526(1.0)	6(1.5)	0.290
Cervical incompetence	56(0.1)	56(0.1)	0	–
Premature birth	3648(6.6)	3615(6.6)	33(8.1)	0.221
Chronic pulmonary disease	1383(2.5)	1366(2.5)	17 (4.2)	< 0.05^*^
Hypertension	879(1.6)	860(1.6)	19(4.7)	< 0.001^**^
Hypertension-complicated pregnancy	1151(2.1)	1140(2.1)	11(2.7)	0.381
Diabetes complicating pregnancy childbirth	2655(4.8)	2636(4.8)	19(4.7)	0.895
Hyperlipidemia	321(0.6)	314(0.6)	7(1.7)	< 0.05^*^
Heart disease	1551(2.8)	1517(2.8)	34(8.4)	< 0.001^**^
Anemia	5872(10.7)	5824(10.7)	48(11.8)	0.446
Cerebrovascular disease	250(0.5)	240(0.4)	10(2.5)	< 0.001^**^
Parkinson disease	24(0.04)	20(0.03)	4(1.0)	< 0.001^**^
Epilepsy	112(0.2)	108(0.2)	4(1.0)	< 0.001^**^
Tuberculosis	59(0.1)	59(0.1)	0	–
Asthma	892(1.6)	883(1.6)	9(2.2)	0.338
Chronic kidney disease	145(0.3)	140(0.3)	5(1.2)	< 0.001^**^

^a.^*p*-values are two-sided

^b^ TWD refers to New Taiwan dollars, of which 1 US dollar = 30TWD

^**^p < 0.001, ^*^p < 0.05

### Multivariable logistic regression analysis of postpartum depression

Table 2 shows the logistic regression analysis results for certain risk factors of PPD. A monthly income of less than NT$ 20,000 and aged 20–24 were used as the reference. Heart disease (aOR 1.95 (1.26, 3.02), *p* < 0.001), antepartum UTI (aOR 1.27 (1.07, 1.65), *p* < 0.05), and age≧40 (aOR 1.98 (1.20, 3.29), *p* < 0.01) were all significant risk factors of PPD.

**Table 2:** The association between PPD and certain risk factors (in which total n≧5 in the PPD group) analyzed by multivariate logistic regression model

	Crude odds ratio	95% Confidence Interval	Adjusted odds ratio	95% Confidence Interval
Antepartum UTI	1.36	(1.05, 1.76)^a^	1.27	(1.07, 1.65)^a^
Upper UTI	3.28	(1.45, 7.41)^b^	2.97	(1.31, 6.77)^b^
Lower UTI	1.29	(1.04, 1.68)^a^	1.21	(1.02, 1.58)^a^
Age group (reference: 20–24)				
20–24	1		1	
25–29	1.00	(0.74, 1.37)	1.05	(0.77, 1.43)
30–34	0.89	(0.65, 1.22)	0.97	(0.71, 1.34)
35–39	0.92	(0.62, 1.36)	1.02	(0.69, 1.53)
≧40	2.89	(1.90, 4.40)^c^	1.98	(1.20, 3.29)^b^
Monthly income (TWD) (reference:< 20,000)				
20,000–40,000	0.82	(0.65, 1.03)	0.86	(0.68, 1.09)
40,000–60,000	0.57	(0.35, 0.93)^a^	0.59	(0.36, 0.97)^a^
≧60,000	0.26	(0.37, 1.86)	0.25	(0.04, 1.81)
Co-morbidity disease				
Chronic pulmonary disease	1.71	(1.05, 2.78)^a^	0.87	(0.50, 1.53)
Hypertension	3.07	(1.93, 4.89)^c^	1.11	(0.61, 2.04)
Hyperlipidemia	3.04	(1.43, 6.47)^b^	1.20	(0.52, 2.75)
Heart disease	3.20	(2.25, 4.57)^c^	1.95	(1.26, 3.02)^b^
Cerebrovascular disease	5.73	(3.02, 10.87)^c^	1.36	(0.58, 3.19)
Chronic kidney disease	4.86	(1.98, 11.92)^b^	1.19	(0.42, 3.38)

Note 1. a: *p* < 0.05, b: *p* < 0.01, c: *p* < 0.001

Note 2. TWD refers to New Taiwan dollars, of which 1 US dollar = 30 TWD

Among all antepartum UTIs, upper tract UTIs (aOR 2.97 (1.31,6.77)) had a higher OR than lower tract UTIs (aOR 1.21 (1.02, 1.58)). In addition, the aOR of monthly income NT$40,000–60,000 was 0.59 ((0.36, 0.97), *p* < 0.05).

## Discussion

This is the first study that has focused on the relationship between antepartum UTIs and PPD. The results of this 14-year population based study show that antepartum UTIs are significantly associated with PPD. In addition, women with upper antepartum UTIs had near 3-fold greater risk of PPD than normal groups (aOR 2.97(1.31, 6.77)).

According to the study results, chronic kidney disease, epilepsy, heart disease, and upper UTI are the four greatest risk factors for PPD. These findings are consistent with those of previous studies that have also found that chronic kidney disease, epilepsy, and heart disease are associated with major depressive disorder or PPD [22–28]. In fact, the relationship between chronic kidney disease and major depressive disorder is well documented [22–24], with the decreased quality of life caused by chronic kidney disease having been found to affect mental health in both men and women [24]. Similarly, the relationship between epilepsy and higher rates of PPD is well documented [25–27]. Meanwhile, a prospective study revealed that the rate of neonatal complications is significantly higher in women with heart disease [28]. Therefore, it is reasonable that PPD is related to antepartum heart disease.

UTIs are among the most common bacterial infections in women, affecting approximately 10% of women. A previous study showed that 2–10% of pregnant women had suffered from a UTI [8], while another study found that 10% of all antepartum admissions were due to UTIs [29]. The higher UTI rate is caused by anatomical and physiological changes to the urinary tract system due to pregnancy [30]. Furthermore, pregnancy also causes asymptomatic bacteriuria changes to symptomatic UTI and reinfection [31].

The symptoms accompanying a UTI include urinary frequency and urgency, painful urination, and lower abdomen discomfort. Overactive bladder syndrome shares some of the same symptoms as UTIs, including urinary frequency and urgency, as well as urgency incontinence. In non-pregnant women, an association between overactive bladder syndrome and major depressive disorder has previously been reported [32–36]. Relatedly, van de Pol et al. demonstrated a significant relationship between PPD and overactive bladder syndrome [37]. Pregnant women who experience urinary frequency and urgency due to a UTI or overactive bladder syndrome may be embarrassed to talk about these symptoms with their physicians, which may further increase the mental stress experienced by these women. Abdollahi et al. demonstrated that recurrent UTIs are associated with PPD, but they did not further examine the relationship with respect to upper and lower UTIs [38].

Antepartum UTIs and PPD have several common risk factors such as obstetric, pediatric, and socioeconomic factors. Mazor-Dray et al. demonstrated that maternal UTIs are associated with preterm delivery, preeclampsia, intra-uterine growth restriction, and cesarean delivery [12]. Furthermore, the rates of PPD have been found to be as high as 40% among women with premature delivery [39], while Yang et al. demonstrated that cesarean delivery, preterm delivery, and preeclampsia were associated with the development of PPD [21]. That being said, preeclampsia was not shown to be associated with PPD in our study. Otherwise, women of low socioeconomic status have a five-fold greater incidence of bacteriuria than normal populations. Low socioeconomic status has also been found to be a risk factor for both antepartum UTIs [40, 41] and PPD [42, 43], while antepartum UTIs are an independent risk factor of PPD.

In our study, upper antepartum UTIs had a higher aOR (OR 2.97 (1.31,6.77)) than lower UTIs. Upper antepartum UTIs are more common during the second half of pregnancy [44], are the most common severe complication of pregnancy [28], and cause approximately 12% of pregnancy associated sepsis complications that result in admission to an obstetric intensive care unit [45]. Approximately 20% of upper UTIs in pregnant women will lead to sepsis and multiple-system failure from endotoxemia [44, 46]. Upper antepartum UTIs reduce physical strength and increase mental stress in pregnant women. In addition, upper antepartum UTIs has 2.97-fold risk of preterm delivery [47], which is another important risk factor of PPD [21].

This study was a retrospective population-based study. Some data that can be useful in determining the severity of a UTI, such as the bacterial species involved, laboratory findings, asymptomatic bacteriuria status, and the side of pyelonephritis, were not available in the NHIRD. In addition, the number of upper and lower antepartum UTI patients in the study was relatively limited. Moreover, we collected PPD patients by coding ICD-9-CM coded which were diagnosed by psychiatrists who have individual concerns about PPD diagnosis. The incidence of PPD in the study cohort may have been underestimated as some women who experience depression after delivery do not seek medical help. Large-scale, prospective studies are needed to further evaluate the association between antepartum UTIs and PPD.

## Conclusions

The results of this study demonstrate that antepartum UTIs, particularly upper antepartum UTIs, are significantly associated with PPD. Specifically, women with upper antepartum UTIs had 2.97-fold increased risk of PPD. As UTIs are among the most common bacterial infections in pregnant women, our results provide useful information for gynecologists, obstetricians, and health policy planners. Above all, they indicate that these health care professionals should pay greater attention to the mental health of mothers who have suffered upper UTIs during their pregnancies.

## Abbreviations

CCI: Charlson comorbidity index
ICD-9-CM: International classification of diseases, 9th revision, clinical modification
LHID 2000: Longitudinal health insurance database 2000
NHIRD: National health insurance research database
OR: Odds ratio
PPD: Postpartum depression
UTI: Urinary tract infection
